# Uterine Arteriovenous Malformation Associated With Retained Products of Conception Treated by Selective Arterial Embolization: A Case Report

**DOI:** 10.7759/cureus.110646

**Published:** 2026-06-11

**Authors:** Oumaima Damoun, Kawtar Cherradi, Samir Bargach

**Affiliations:** 1 Department of Gynecology-Obstetrics and High Risk Pregnancy, Maternity Souissi Hospital Center Ibn Sina, University Mohamed 5, Rabat, MAR

**Keywords:** doppler ultrasound, pelvic magnetic resonance imaging, retained products of conception, uterine arteriovenous malformation, uterine artery embolization

## Abstract

Uterine arteriovenous malformation is an uncommon but potentially life-threatening cause of abnormal uterine bleeding, particularly after pregnancy-related intrauterine instrumentation. It can mimic or coexist with retained products of conception, and inappropriate curettage may precipitate severe hemorrhage.

We report the case of a 27-year-old G1P0 woman who presented with recurrent metrorrhagia, pelvic pain, and two months without a normal menstrual period, five months after an unsupervised termination of pregnancy followed by uterine curettage. She was hemodynamically stable but anemic, with a hemoglobin level of 8.9 g/dL, and serum beta-human chorionic gonadotropin was negative. Transvaginal ultrasonography with color Doppler showed intracavitary material and serpiginous endometrial-myometrial hypervascular channels with turbulent flow. Pelvic magnetic resonance imaging demonstrated posterior myometrial flow voids with early intense enhancement and a dominant arterial supply from the left uterine artery. Pelvic angiography confirmed a high-flow arteriovenous shunt supplied predominantly by the left uterine artery. Selective arterial embolization using absorbable gelatin sponge particles achieved immediate angiographic occlusion of the shunt and clinical control of bleeding. Follow-up ultrasonography at one month showed marked regression, and a six-month examination confirmed complete resolution without recurrent bleeding.

This case underscores the importance of considering a uterine vascular lesion in reproductive-age patients with persistent bleeding following pregnancy loss or curettage. Multimodal imaging is essential for distinguishing vascular retained products of conception, enhanced myometrial vascularity, pseudoaneurysm, and uterine arteriovenous malformation. In hemodynamically stable patients desiring future fertility, selective embolization represents an effective fertility-preserving treatment option that avoids the hemorrhagic risks associated with blind curettage.

## Introduction

Uterine arteriovenous malformation (UAVM) is a rare vascular lesion characterized by abnormal communications between uterine arterial and venous channels without an intervening capillary bed. Although uncommon, it is clinically important because it may present with recurrent abnormal uterine bleeding or sudden severe hemorrhage in women of reproductive age [1,2].

Most clinically recognized UAVMs are acquired rather than congenital. Reported triggers include dilatation and curettage, cesarean delivery, uterine surgery, gestational trophoblastic disease, and pregnancy loss. Post-pregnancy vascular lesions may overlap in terminology and appearance with enhanced myometrial vascularity (EMV) and vascular retained products of conception (RPOC), which makes precise preoperative diagnosis challenging [3-6].

Transvaginal ultrasonography with color and spectral Doppler is generally the first-line imaging test. Magnetic resonance imaging (MRI) helps define the myometrial extent, flow voids, feeding vessels, and relationship with intracavitary material, while digital subtraction angiography can confirm the diagnosis and provide a therapeutic route [1,2]. Recognition is essential because blind curettage in an unrecognized high-flow lesion can worsen bleeding. We present a case of an acquired UAVM associated with suspected RPOC after curettage, successfully managed with selective arterial embolization [7,8].

## Case presentation

A 27-year-old G1P0 woman presented with recurrent moderate metrorrhagia and pelvic pain following 2 months of amenorrhea. These symptoms had been occurring intermittently for the preceding month. Her gynecologic history was notable for an unsupervised termination of pregnancy followed by uterine curettage five months before presentation. She had no reported medical, surgical, or thromboembolic history.

On examination, she was in good general condition and hemodynamically stable. Abdominal palpation revealed mild pelvic tenderness. Speculum examination confirmed bleeding from an intrauterine source, with no visible cervical lesion. Initial laboratory testing showed moderate anemia, with a hemoglobin level of 8.9 g/dL. Serum beta-human chorionic gonadotropin was negative, and coagulation parameters were within normal limits.

The clinical timeline was as follows: pregnancy termination and curettage occurred five months before presentation; intermittent bleeding and pelvic pain developed during the month before admission; ultrasonography and MRI were performed during the diagnostic workup; angiography with embolization was performed after noninvasive imaging identified a high-flow uterine vascular lesion; ultrasound follow-up was obtained at one and six months.

Transvaginal ultrasonography with color and pulsed Doppler showed a left-sided intracavitary lesion extending from the endometrium toward the myometrium. It contained tortuous hypervascular channels with turbulent flow, raising concern for a uterine arteriovenous malformation associated with suspected retained products of conception or blood products (Figure 1).

**Figure 1:**
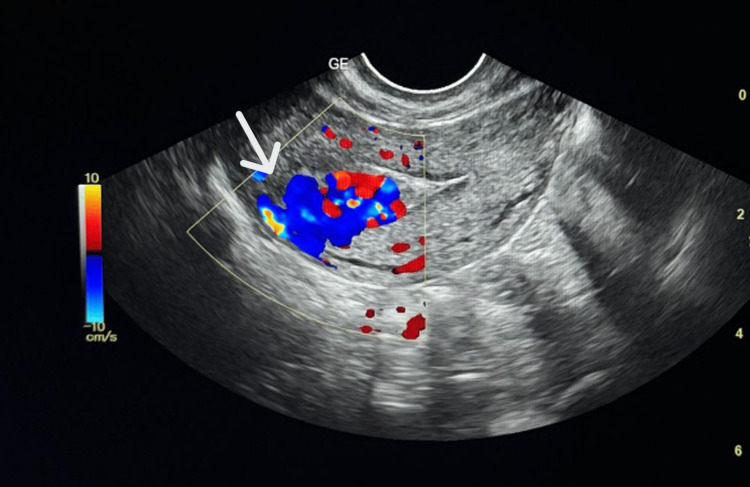
Transvaginal color Doppler ultrasonography showing serpiginous endometrial-myometrial vascular channels with turbulent flow (white arrow)

Pelvic MRI showed an anteverted and anteflexed uterus of normal size, measuring 115 x 60 x 36 mm, with endometrial intracavitary material. Serpiginous posterior myometrial flow voids were seen, with early intense post-contrast enhancement and apparent communication with the left uterine artery. There was no diffusion restriction. Blood products were also present within the vaginal canal. These findings supported an acquired high-flow uterine vascular lesion with dominant left uterine arterial supply (Figure 2).

**Figure 2:**
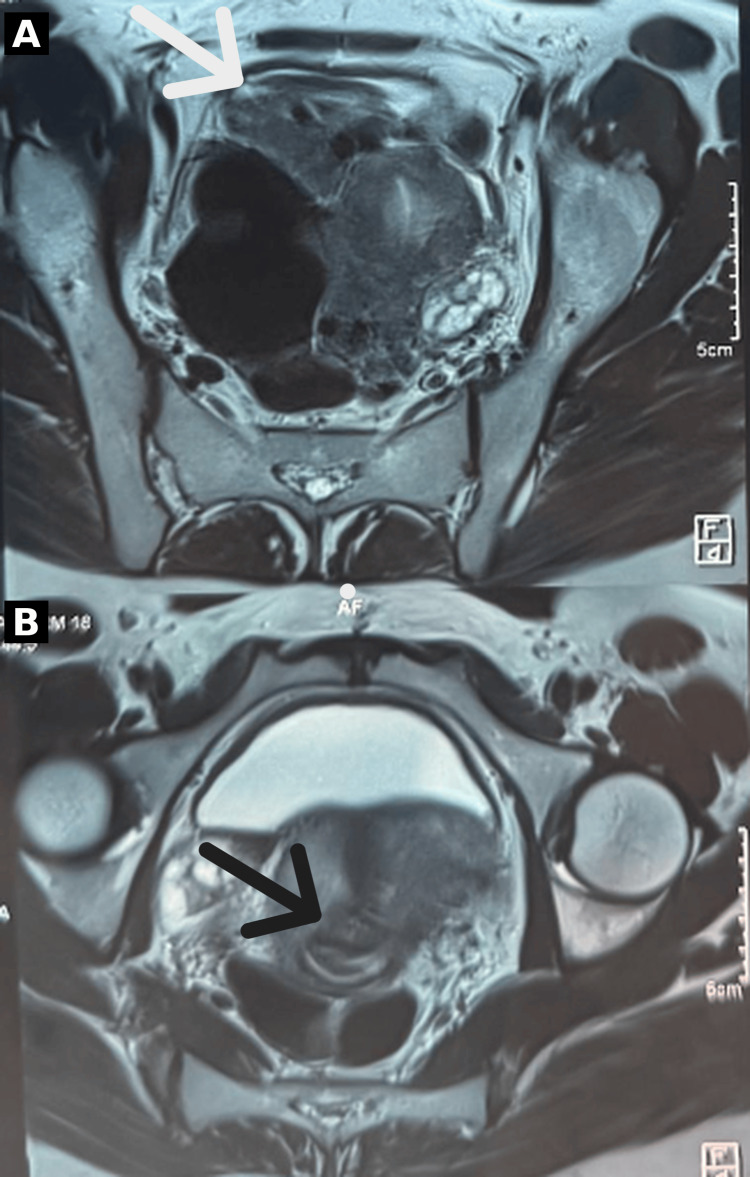
Pelvic magnetic resonance imaging Panel A shows serpiginous myometrial vascular structures in the left posterior uterine wall (white arrow). Panel B shows intracavitary material and adjacent pelvic anatomy (black arrow).

Because of the persistent bleeding and high-flow imaging pattern, pelvic angiography was performed for diagnostic confirmation and treatment. Angiography demonstrated marked dilation of the left uterine artery, irregular uterine arterial ramifications, early venous opacification, and a corkscrew appearance of the feeding branches, confirming an arteriovenous shunt supplied predominantly by the left uterine artery.

Selective arterial embolization was performed using absorbable gelatin sponge particles (Curaspon; Cura Medical, Assendelft, The Netherlands) injected until angiographic exclusion of the shunt was achieved. The procedure was uncomplicated. Final angiography confirmed occlusion of the arteriovenous shunt with preservation of the main vascular axes.

The patient was observed during a short hospital stay, and the postoperative course was uneventful. Ultrasound at one month showed marked regression of the vascular lesion and disappearance of the intracavitary retention-like material. At six months, transvaginal ultrasonography confirmed complete resolution of the vascular abnormality, with a thin regular endometrium and no recurrent hemorrhage (Figure 3). The patient reported the return of regular menstrual cycles.

**Figure 3:**
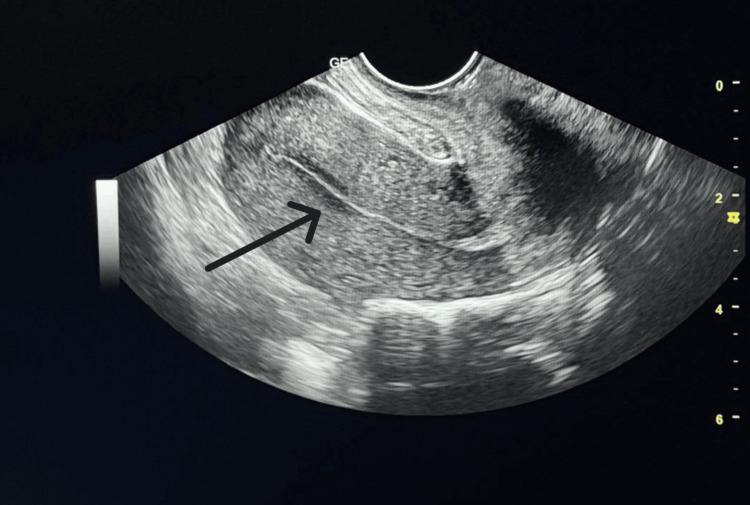
Follow-up transvaginal ultrasonography at six months showing an empty uterine cavity with a thin regular endometrium (black arrow)

## Discussion

This case illustrates several practical points in the evaluation of abnormal uterine bleeding after pregnancy-related instrumentation. The combination of recent curettage, recurrent bleeding, anemia, and a high-flow Doppler pattern should prompt consideration of UAVM, EMV, pseudoaneurysm, and vascular RPOC before any repeat intrauterine procedure is attempted [4-8].

The terminology in this field is important. A true acquired UAVM implies an arteriovenous shunt, whereas EMV is a sonographic description of increased myometrial vascularity that may be seen with RPOC, subinvolution of the placental bed, or other post-pregnancy conditions [4,5]. In our patient, angiography demonstrated early venous drainage through a high-flow shunt, supporting the diagnosis of acquired UAVM. However, the associated intracavitary material was not sampled histologically because repeat curettage was intentionally avoided. Therefore, the diagnosis of RPOC should be interpreted as an imaging-based diagnosis rather than a pathologic confirmation.

The negative serum beta-human chorionic gonadotropin level also argues against active gestational trophoblastic neoplasia, although it does not completely exclude devitalized retained tissue or post-procedural blood products. In similar scenarios, serial beta-human chorionic gonadotropin testing, Doppler evaluation of peak systolic velocity when available, MRI, and angiography can help clarify the risk profile and guide management [4-8].

Management should be individualized according to hemodynamic status, severity of bleeding, flow characteristics, fertility goals, and local expertise. Observation may be reasonable for selected stable patients with low-flow EMV and declining symptoms. Hysteroscopic or surgical treatment can be considered for avascular or low-risk RPOC. In contrast, symptomatic high-flow lesions are commonly treated with uterine artery embolization because it can rapidly control bleeding while preserving the uterus [1,2,9-12]. A systematic review reported a high overall success rate for uterine artery embolization in UAVM, with subsequent pregnancies documented after treatment, although reintervention can be required after incomplete embolization or recurrence [10].

The embolic material should be chosen according to lesion anatomy, flow dynamics, operator experience, and the need to preserve fertility. Absorbable gelatin sponge can provide temporary occlusion and was effective in this case. Other agents, including coils, particles, glue, or combinations, have also been used in reported cases [9-15]. Potential complications include pelvic pain, fever, non-target embolization, uterine ischemia, and, rarely, thromboembolic events. These risks support treatment in centers with interventional radiology expertise and multidisciplinary coordination among gynecology, radiology, and anesthesia teams.

It is essential to emphasize that embolization is an invasive and irradiating procedure, requiring experienced interventional radiologists and an appropriate technical facility. Furthermore, although its advantages over radical surgery are undeniable, a few uncommon and non-specific complications exist regarding UAVMs. Most frequently, these are minor complications, such as transient pelvic pain and fever. However, severe-albeit extremely rare-complications have been reported; these are primarily associated with internal iliac artery embolization, which can lead to skin necrosis, neurological deficits, and recto-vesico-vaginal fistulas [16]. Although rare, pulmonary embolism also constitutes a potential risk in the treatment of UAVMs via uterine artery embolization, as embolic particles introduced during catheterization can accidentally enter the systemic venous system and migrate into the pulmonary circulation, thereby inducing a pulmonary embolism [17].

Our case illustrates the paramount importance of early clinical suspicion when confronted with recurrent post-traumatic vaginal bleeding, as well as the added value of a targeted endovascular diagnostic and therapeutic approach. A multidisciplinary management strategy, combining gynecologists, interventional radiologists, and anesthesiologists, remains the key to optimizing both the functional and reproductive prognoses of patients presenting with UAVM.

## Conclusions

Uterine arteriovenous malformation should be considered in reproductive-age patients with persistent or recurrent uterine bleeding after pregnancy loss, curettage, or other intrauterine procedures. Color Doppler ultrasonography and MRI can identify high-flow vascular patterns and intracavitary material, while angiography confirms the shunt and enables treatment. In hemodynamically stable patients with fertility-preservation goals, selective arterial embolization offers effective hemostasis and avoids the potential hemorrhagic danger of blind curettage. Accurate terminology, careful exclusion of vascular products of conception and enhanced myometrial vascularity, and multidisciplinary planning are essential for safe management.
